# Controllable optical activity with non-chiral plasmonic metasurfaces

**DOI:** 10.1038/lsa.2016.96

**Published:** 2016-07-01

**Authors:** Ping Yu, Jianxiong Li, Chengchun Tang, Hua Cheng, Zhaocheng Liu, Zhancheng Li, Zhe Liu, Changzhi Gu, Junjie Li, Shuqi Chen, Jianguo Tian

**Affiliations:** 1 The MOE Key Laboratory of Weak Light Nonlinear Photonics, School of Physics, Teda Applied Physics Institute, and the 2011 Project Collaborative Innovation Center for Biological Therapy, Nankai University, Tianjin 300071, China; 2 Beijing National Laboratory for Condensed Matter Physics, Institute of Physics, Chinese Academy of Sciences, Beijing 100190, China

**Keywords:** metasurfaces, optical activity, surface plasmon polaritons

## Abstract

Optical activity is the rotation of the plane of linearly polarized light along the propagation direction as the light travels through optically active materials. In existing methods, the strength of the optical activity is determined by the chirality of the materials, which is difficult to control quantitatively. Here we numerically and experimentally investigated an alternative approach to realize and control the optical activity with non-chiral plasmonic metasurfaces. Through judicious design of the structural units of the metasurfaces, the right and left circular polarization components of the linearly polarized light have different phase retardations after transmitting through the metasurfaces, leading to large optical activity. Moreover, the strength of the optical activity can be easily and accurately tuned by directly adjusting the phase difference. The proposed approach based on non-chiral plasmonic metasurfaces exhibits large optical activity with a high controllable degree of freedom, which may provide more possibilities for applications in photonics.

## Introduction

Optical activity is the rotation of the plane of linearly polarized (LP) light along the propagation direction as the light travels through optically active materials, and it has acquired considerable importance in analytical chemistry, spectroscopy, crystallography and molecular biology^1, 2^. Optical activity is a type of birefringence, and it occurs in solutions of chiral molecules such as sucrose, solids with rotated crystal planes such as quartz, and spin-polarized gases of atoms or molecules. The molecular structures of these materials exhibit a geometry where the original structure cannot be made congruent with its mirror image^3^. When LP light is transmitted through optically active materials, the right and left circular polarization components of the LP light experience different refractive indexes, leading to a change in the relative phase between the two circular polarizations. The polarization of the transmitted light is thus rotated at an angle^4, 5^. The degree of rotation depends on the propagation length in the material and the specific rotation, which is proportional to the refractive index difference (Δ*n*). For conventional optically active materials, Δ*n* is related to the specific crystal system of the birefringent crystal. Therefore, the strength of optical activity can be controlled by tuning the molecule arrangements of special optically active materials, such as liquid crystals and magneto-optical crystals.

Metamaterials are artificial media on a size scale smaller than the wavelength of incident light, and the artificial structural units function as atoms and molecules in conventional materials^6, 7, 8^. Through regulated interactions with electromagnetic waves, metamaterials can produce remarkable physical properties unavailable in naturally occurring or chemically synthesized materials^9, 10, 11, 12, 13^. By designing structural units with chirality, metamaterials can also generate large optical activity with a mechanism similar to that in traditional optically active materials^14, 15, 16, 17, 18^. Recently, plasmonic metasurfaces have been introduced, which are metamaterials with reduced dimensionality, typically consisting of a two-dimensional arrays of nanostructures^19, 20, 21, 22, 23, 24^. Plasmonic metasurfaces are capable of controlling the optical phase of transmitted or reflected light at the nanoscale and generating a wide range of position-dependent discontinuous interfacial phase profiles. Research interests have moved toward fascinating phenomena and applications, such as anomalous refraction or reflection^19, 25, 26, 27, 28^, vortex or vector beam generation^29, 30, 31^ and wave plates^32, 33, 34, 35, 36, 37, 38^. Because plasmonic metasurfaces enable unprecedented degrees of freedom in light phase manipulation, they can be used to directly design the transmitted phase of right circularly polarized (RCP) and left circularly polarized (LCP) light. Optical activity can thus also be realized under normal incidence through another efficient method in which the chirality of the nanostructures is not a critical factor. Therefore, metasurfaces provide a feasible method for realizing and controlling optical activity via directly designing ‘atoms’ or ‘molecules’ in non-chiral plasmonic metasurfaces.

Here we propose an alternative approach to realize controllable optical activity using single-layer, non-chiral, plasmonic metasurfaces composed of cross-shaped nanoaperture arrays. Through accurate design of the structural units of the non-chiral metasurfaces, the incident LP light can be converted into LCP and RCP beams on a nanoscale. The phase retardation difference between the LCP and RCP beams can be easily modulated by varying the geometrical parameters of the nanoapertures, leading to continuously controllable optical activity. We theoretically analyzed the physical mechanisms and numerically and experimentally proved the theory using the designed non-chiral metasurfaces. Compared with metamaterials with chirality, the proposed non-chiral metasurfaces could more directly manipulate the phase retardation, which provides another efficient method for realizing optical activity.

## Materials and methods

### Numerical simulations

All numerical simulations were performed using the commercial software COMSOL Multiphysics based on the finite element method. The permittivity of silver is defined by the Drude model 
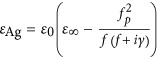
 with 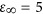, *f*_*p*_=2.175 PHz and *γ*=4.35 THz^39^. We chose silver because it performs well in manipulating the light phase compared with other metals, such as gold. The refractive index of the glass substrate is taken as 1.47. For the theoretical simulations, the unit cell has periodic boundary conditions in the *x* and *y* planes, and the waveguide ports boundary conditions on other boundaries.

### Measurement setup for optical activity

**Figure 1:**
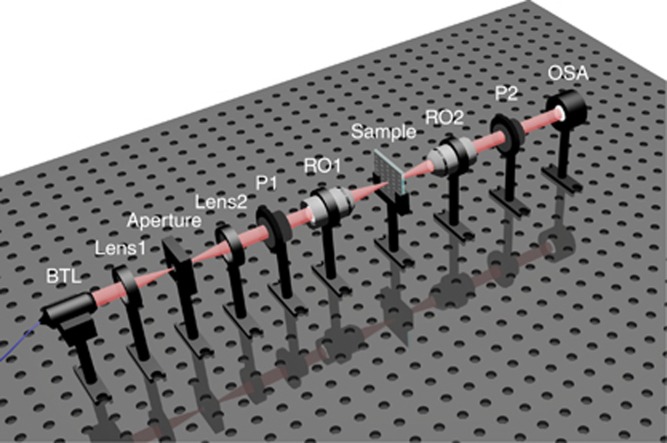
White light is generated by a BTL. The polarization direction of incident light is controlled by polarizer P1. As the angle of another polarizer P2 is rotated, the transmission through the sample is collected by the OSA. Two reflective objectives (RO1 and RO2) are used to focus and collect the light. BTL, bromine tungsten lamp; OSA, optical spectrum analyzer.

The experiments were performed using a custom-built optical setting, as shown in Figure 1. White light from a bromine tungsten lamp in the normal direction was used as the light source. The spot size of the incident light needs to be adjusted according to the size of the samples in the experimental process. Two lenses form a 4f-system. The aperture is set near the focus of the 4f-system. The sample can be sufficiently illuminated by the incident light by adjusting the size of the aperture. A polarizer (P1) was used to control the normal incident LP light polarized along *θ*=45°, which was focused on the sample with a reflective objective (RO1). The transmission through the sample was collected using another similar reflective objective (RO2). Another polarizer (P2) was inserted into the optical path to analyze the polarization status of the transmitted light. Optical transmission was collected by an optical spectrum analyzer via a fiber coupler, which enabled verification of the polarization rotation phenomenon generated by the sample. By rotating the angle of the analyzer within a range of 180°, we could obtain the polarization state of the transmitted light over a broad wavelength range. All optical elements, including the microscope objective, lens, polarizers and detector, were operated in the broadband range.

### Sample fabrication

Sputtering deposition, electron-beam lithography and reactive-ion etching were used to fabricate the metallic structures. The samples for generating different optical rotation were fabricated as follows. Silver was deposited onto the quartz plate to a thickness of 35 nm using a radio-frequency magnetic sputtering system, and a 100-nm-thick PMMA resist was subsequently spin-coated onto the sample. The sample was then subjected to bakeout at 120 °C on a hotplate for 1 min. The cross-shaped pattern was exposed by the direct line writing method with an electron-beam lithography system (JEOL, JBX6300FS) at 100 keV and 3000 μC cm^−1^. After exposure, the sample was developed in MIBK:IPA (1:3) for 40 s and IPA for 30 s and was then blown dry using pure nitrogen. Finally, the pattern was transferred onto the silver layer by Ar plasma reactive-ion etching at a power of 100 W, a flow rate of 40 sccm and a pressure of 30 mTorr. The remaining PMMA resist was removed using hot acetone at 60 °C for 30 min.

## Results and discussion

### Theoretical analysis

Any LP light can be written as an equal combination of RCP and LCP light^40^:


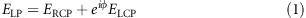


where *E* is the electric field of the light. The phase difference *φ* between the two circular polarizations governs the direction of the linear polarization. The optical activity is the rotation of LP light by adjusting *φ*. In contrast to existing optically active materials with chirality, the non-chiral plasmonic metasurface employed in our novel method enables direct modulation of the phase difference *φ* and thus enables controllable optical activity. The process of optical activity generation involves two steps: (i) conversion of the LP incident light into RCP and LCP beams and (ii) modulation of the phase difference of the LCP and RCP beams, and generation of new LP light with rotated plane due to local coupling.

We first focus on the generation of the RCP and LCP beams. The Dirac bracket notation is adopted to define the light polarization state. When LP light 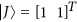 is normally incident on the non-chiral metasurface, the interaction between the light and the metasurface can be expressed as^41, 42^


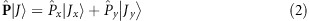


where 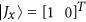 and 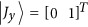 are two orthogonal components in the *x* and *y* directions of the incident LP light, respectively. The operator 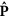 represents the influence of the metasurface on the amplitude transmission and phase change of the incident light. The two orthogonal components of 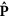 can be expressed as


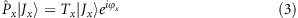



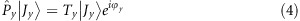


where *T*_*m*_ and 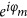 (*m*=*x*, *y*) denote the amplitude transmittance and phase factor of the transmitted beams in the *m* direction. We replace the LP light with two opposite-helicity circularly polarized (CP) beams to simplify the mechanism analysis. By substituting Equations (3) and (4) into Equation (2) and applying the replacement 
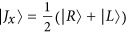
 and 
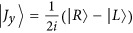
, Equation (2) can be rewritten as


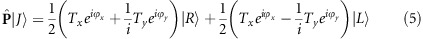


where 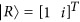 and 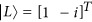 correspond to the RCP and LCP beams, respectively. According to Equation (5), if *T*_*x*_*=T*_*y*_, RCP or LCP light can be obtained when the phase difference satisfies 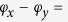 −*π*/2 or *π*/2. Thus, we can convert the LP incident light into RCP or LCP light by designing different nanostructures. The expressions of two generated CP beams can be simplified as 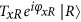 and 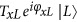, where *T*_*xn*_ and *ϕ*_*xn*_ (*n*=*R*, *L*) represent the transmission amplitude and phase of the RCP and LCP beams along the *x*-axis.

The LCP and RCP beams need to be modulated to generate a phase difference 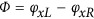 before they are superposed into new LP light. The combination process of the RCP and LCP beams can be written as:


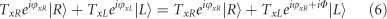


Assuming the transmission amplitudes of RCP and LCP waves have the same value *T*_*xR*_=*T*_*xL*_, we can obtain the transmitted light with a simple form, as follows:


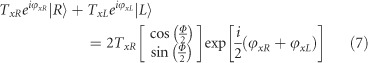


Equation (7) shows that the transmitted light exhibits linear polarization, and the polarization direction is governed by *Φ*/2.

### Sub-units of non-chiral metasurfaces

**Figure 2:**
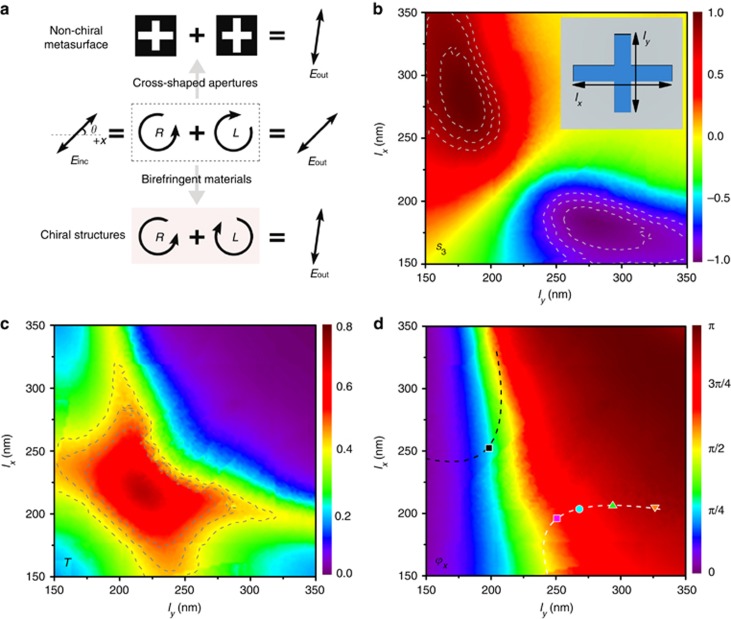
(**a**) Theory of controllable optical activity (middle region). Top and bottom regions indicate the optical rotations with non-chiral plasmonic metasurfaces and conventional methods, respectively. *θ* is the angle between the incident light and the *x*-axis. (**b**) Normalized Stokes parameter *S*_3_, (**c**) transmission *T* and (**d**) phase *ϕ*_*x*_ in the *x*-direction as functions of *l*_*x*_ and *l*_*y*_, which correspond to the lengths of the cross-shaped nanoaperture, as shown in the inset of **b**. The periods in the *x* and *y* directions are *l*_*x*_+100 nm and *l*_*y*_+100 nm, respectively.

Based on the characteristics of flexible phase modulation, the process described above for optical activity generation can be achieved using non-chiral plasmonic metasurfaces. As shown in Figure 2a, the controllable optical activity arises from the phase control of the right and left circular polarization components decomposed from the LP light. Compared with the conventional method of achieving optical activity using birefringent materials, we utilized a cross-shaped nanoaperture as the sub-unit because it can be easily designed for the conversion of LP light into CP light^43^. Two different types of cross-shaped nanoapertures that could generate RCP and LCP light were combined, thus forming a unit cell of the proposed non-chiral plasmonic metasurface. The relative phase retardation between two opposite-helicity CP beams can be tuned by directly changing the geometrical parameters of the nanoapertures while removing the additional external conditions, such as the external electric or magnetic fields. It is therefore possible to simultaneously realize and control the optical activity by designing the geometrical parameters of the two sub-units of the cross-shaped nanoapertures.

We now aim at tuning the lengths of the cross-shaped nanoaperture to find the specific sub-units that can satisfy the requirements of the theory. A 35-nm-thick silver film with cross-shaped nanoapertures is illuminated by the LP incident plane wave at a wavelength of 990 nm and exhibits polarization along the direction *θ*=45°. The corresponding sub-unit is shown in the inset of Figure 2b. The theoretical simulations are studied by the finite element method using COMSOL Multiphysics (see the Materials and Methods section)^44^. As mentioned above, the first step of realizing optical activity is designing the nanostructures for the conversion of LP light into RCP and LCP light. Figure 2b illustrates the normalized Stokes parameter *S*_3_ as a function of the two lengths *l*_*x*_ and *l*_*y*_ of the cross-shaped nanoaperture. A value of *S*_3_ close to 1 or −1 represents nearly perfect RCP or LCP light, respectively, as shown by the red and purple regions in Figure 2b. Two types of sub-units of the non-chiral metasurface should therefore be selected from each region. When placing two sub-units close together, the output LP light is achieved at the far field as a result of the local coupling between the generated RCP and LCP light. The next key issue is the generation of LP light with desired optical activity. Two modulated CP lights with opposite-handedness must have similar amplitude with a relative phase delay *Φ*. Figure 2c and 2d shows the transmission *T* and phase *ϕ*_*x*_ in the *x*-direction of cross-shaped nanoapertures. As illustrated in Figure 2c, the nanoapertures in the region of gray lines have similar *T* values of ~45%. The phase retardation between the LCP and RCP light can be modulated because the transmitted phase strongly depends on the geometrical parameters of the nanoaperture, as shown in Figure 2d. Considering the above results, the sub-units that satisfy the requirements of the theory are indicated by two dashed lines in Figure 2d. R_*j*_ and L_*j*_ are used to represent the sub-units that generate the RCP and LCP light, where *j* is the index of the sub-unit. To simplify the process, we fix the geometrical parameters of sub-unit R_1_, indicated by the black square, and vary the other sub-unit L_*j*_ along the white curve in Figure 2d to vary the phase retardation *Φ* from *π*/2 to *π*. Thus, new LP light with a different degree of optical activity can be realized.

### Process and characteristics of the controllable optical activity

**Figure 3:**
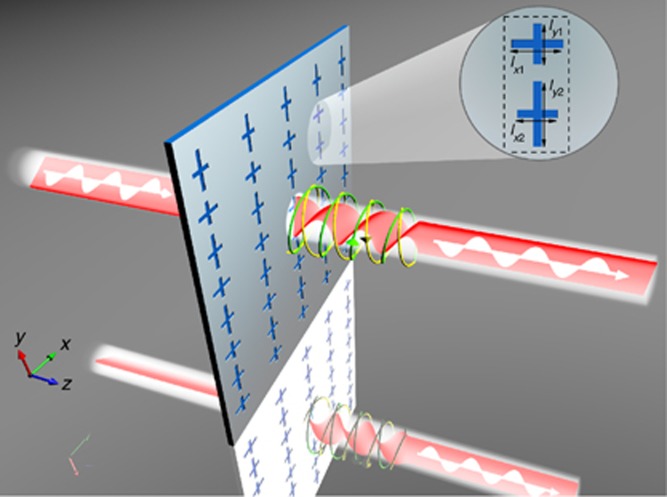
Sketch of the optical activity process. The dashed line in the inset shows the unit cell of the presented non-chiral plasmonic metasurfaces designed with *l*_*x*1_=250 nm and *l*_*y*1_=200 nm; the periods in the *x* and *y* directions are 295 and 680 nm, and all nanoapertures have the same width of 43 nm.

Figure 3 shows the process of realizing the optical activity of LP incident light. A non-chiral plasmonic metasurface is proposed in which each unit cell consists of two sub-units of cross-shaped nanoapertures. These two sub-units split the incident LP beam into two CP beams with opposite-helicity in the near field. These two rays effectively generate new LP light whose polarization has a different direction to that of the incident light. The unit cell of the presented non-chiral metasurface and its parameters are shown in the inset of Figure 3. Due to the coupling between the two sub-units, the periodic lengths of the unit cell have been slightly modified compared with those shown in Figure 2. As mentioned above, we fix the geometrical parameters of the upper cross-shaped nanoaperture R_1_ while varying the *l*_*x*2_ and *l*_*y*2_ of the lower cross-shaped nanoaperture L_*j*_ along the white curve shown in Figure 2d.

**Figure 4:**
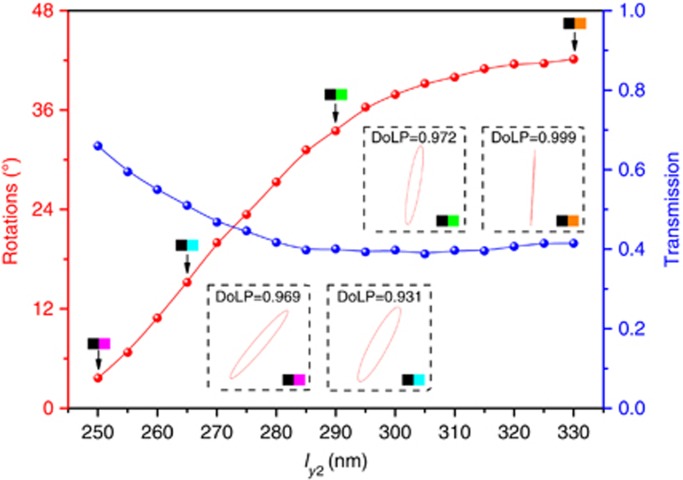
Calculated optical rotations (red line) and amplitude transmissions (blue line) at a wavelength of 990 nm. The parameter *l*_*y*2_ represents the sub-unit L_*j*_ along the white curve shown in Figure 2d. The insets show the polarization states and the corresponding DoLP for four metasurfaces, which correspond to the combinations of R_1_ and L_1_ to L_4_.

The calculated optical rotations and the corresponding transmissions at a wavelength of 990 nm are illustrated in Figure 4 when combing R_1_ and different L_*j*_. The degree of the optical activity effect changes nonlinearly with variation in the geometrical parameters of L_*j*_. The variation in the geometrical parameters generates a relative phase difference *Φ* from ~*π*/2 to *π*. According to Equation (7), optical rotation with arbitrary values from 3° to 42° is obtained. We thus achieved controllable optical activity with relatively high-amplitude transmission beyond 40%. In addition, the transmitted light through each unit cell has high-quality linear polarization. To illustrate this point, we chose four specific structures to calculate the polarization patterns of the transmitted beams, as shown in the insets of Figure 4. The four selected non-chiral metasurfaces correspond to the combinations of sub-unit R_1_ and sub-units L_1_ to L_4_, which are also marked by colorized patterns in the white curve shown in Figure 2d. All of the resultant polarizations are found to be almost linear. The degree of linear polarization (DoLP), defined as 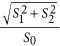, which is represented using Stokes parameters, is also given to effectively show the linear polarization of the transmitted wave. Therefore, the degree of optical activity can be flexibly controlled by appropriately designing the non-chiral plasmonic metasurface without changing the incident wavelength.

**Figure 5:**
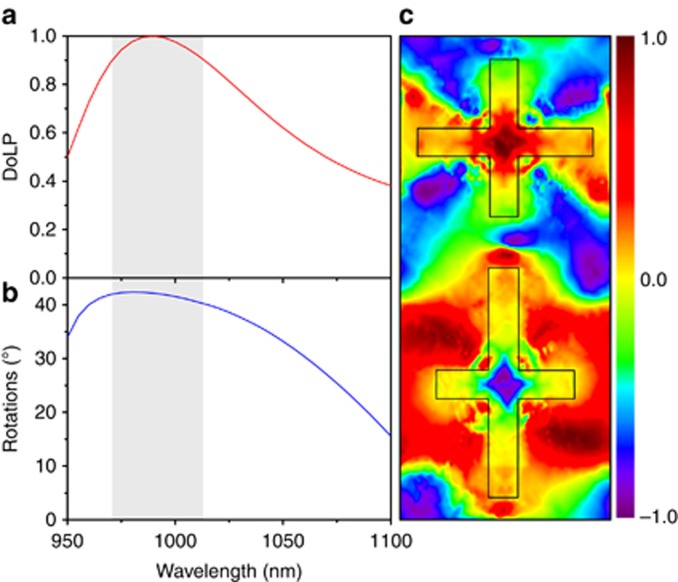
Calculated far-field (**a**) DoLP and (**b**) rotations for the non-chiral metasurface by the combination of R_1_ and L_4_ 900 nm from the exit surface. (**c**) Corresponding near-field normalized Stokes parameters *S*_3_ 5 nm from the exit surface at a wavelength of 990 nm.

To further analyze the properties of the proposed non-chiral metasurface, we calculated the DoLP and the rotations in the broadband range of the non-chiral metasurface with a combination of R_1_ and L_4_, as shown in Figure 5a and 5b. A DoLP value above 0.9 indicated by the shadowed region can be regarded as perfect LP light. The polarization direction of the LP light has an optical rotation of 42° at the far field within the shadowed region. The corresponding normalized Stokes parameter *S*_3_ distribution at 5 nm from the exit surface is shown in Figure 5c. The RCP light is obtained from the upper sub-unit, whereas the LCP light is obtained from the lower sub-unit at the near field. As expected, the results verify the proposed theory that the RCP and LCP light are individually generated at the near field and that new LP light is effectively generated at the far field.

### Measurement of controllable optical activity

**Figure 6:**
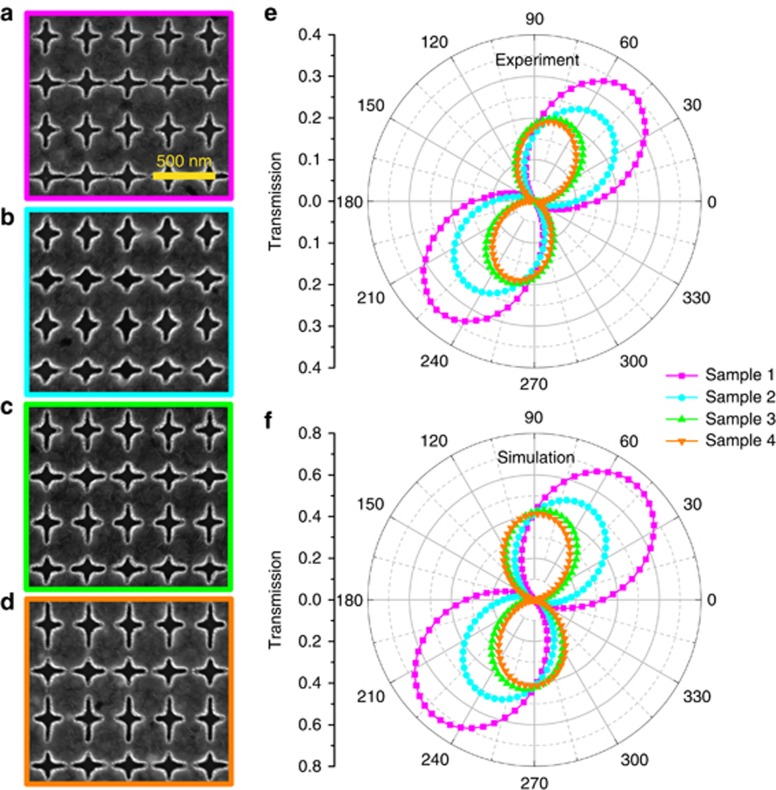
(**a**–**d**) SEM images of the four fabricated non-chiral plasmonic metasurfaces from sample 1 to sample 4, which correspond to the nanostructures by combination of R_1_ and L_1_ to L_4_. (**e**) Experimental and (**f**) numerically simulated transmissions as a function of analyzer angle *ϕ* for the four samples.

Experiments were performed to demonstrate the polarization responses of the designed non-chiral plasmonic metasurfaces. In the experimental process, a polarizer was used to control the polarization direction of the normal incident LP light along *θ*=45°. After passing through the non-chiral metasurfaces, the original polarization direction of incident light is rotated based on different phase delays between the LCP and RCP light. Another polarizer is used to analyze the polarization state of the transmitted light (see the Materials and Methods section). Figure 6a and 6d shows the scanning electron microscopy images of the four non-chiral metasurface samples corresponding to combinations of R_1_ and L_1_ to L_4_. These configurations are experimentally achieved by sputtering deposition of silver film and silicon dioxide and then applying electron-beam lithography followed by reactive-ion etching (see the Materials and Methods section). Figure 6e shows the obtained amplitude transmissions as a function of the analyzer angle *ϕ* while maintaining *θ*=45°. The observed optical rotations Δ, which are characterized by the angle between *ϕ* and *θ*, are 3°, 9°, 23° and 30° for the four samples. All the angles are defined relative to the *x*-axis. We experimentally realized the controllable optical activity at the resonant wavelength of 970 nm by designing different non-chiral metasurfaces. The experimental results are in good agreement with the corresponding numerical results shown in Figure 6f. The slight differences between the experimental and simulated results, such as the degree of rotation and the decrease in the amplitude of transmission, may be caused by the discrepancy of the aperture lengths or the rounding of aperture corners in the fabrication process. In contrast to previous works using intrinsically non-chiral nanostructures (superimposed with their mirror images) under oblique incidence^45, 46, 47, 48^, our proposed method realizes the optical activity with the non-chiral metasurface illuminated by normal incidence.

**Figure 7:**
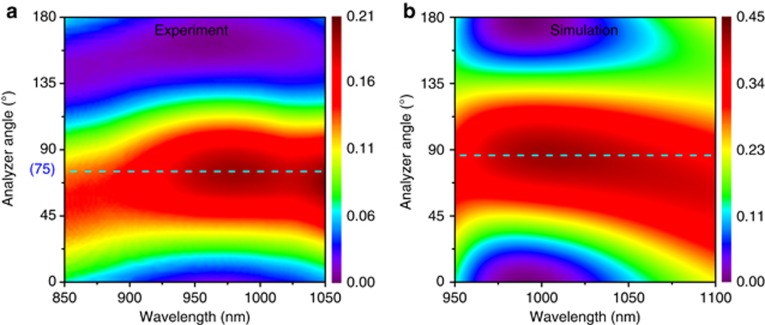
(**a**) Experimentally and (**b**) numerically simulated transmissions as a function of analyzer angle *ϕ* at a broad wavelength range for sample 4.

The experimentally measured transmitted intensity at the broad wavelength range of sample 4 is also shown in Figure 7a. The proposed optical activity with 30° rotation of LP light can be observed at the wavelength range from ~925 to 1025 nm. We also numerically calculated the corresponding transmitted intensity as a function of the analyzer angle *ϕ*, as shown in Figure 7b. The bandwidth of the intensity is almost the same as the corresponding experimental results. The difference is that the experimental resonate wavelength range exhibits a blueshift compared with the simulated results, which may be caused by the thickness difference of the glass substrate between the actual fabrication and the simulations. The proposed controllable optical activity effect can thus also be observed in a broadband wavelength range both numerically and experimentally, which could provide some opportunities in the design of various broadband optical systems and applications.

### General applicability of the controllable optical activity

The proposed approach is not limited only by a certain wavelength range or nanostructure, which has a more general applicability. It is possible to realize a similar optical activity effect at other spectral regimes by changing the geometric parameters or designing other nanostructures. The key factor is that the nanostructures could be easily designed for converting LP light into LCP and RCP light. Significant optical activity can also be achieved by using other non-chiral plasmonic metasurfaces based on the same method. For instance, similar controllable optical activity can be achieved by designing a non-chiral metasurface composed of cross-shaped nanoantennas (see Supplementary Fig. S1 and Supplementary Note 1); the controllable range of optical activity can also be further extended by using a dual-layer, non-chiral, plasmonic metasurface (see Supplementary Figs. S2 and S3 and Supplementary Note 2).

## Conclusions

In conclusion, we present a novel method for realizing optical activity using an ultrathin, non-chiral, plasmonic metasurface consisting of cross-shaped nanoapertures. The physical mechanisms have been analyzed in detail and are different from those of traditional methods that realize optical activity using structures with chirality. We generate the phase delay between LCP and RCP beams by directly designing two different structural units. The optical activity can be flexibly controlled by appropriately varying the non-chiral, optically active, artificial nanostructures. In addition, the controllable property of the optical activity is numerically and experimentally proved, which could provide some potential applications in micro-optical devices and sensors.
