# Supplementary material for: Controllable optical activity with non-chiral plasmonic metasurfaces
Source: Light Sci Appl. 2016 Jul 1;5(7):e16096–. doi: 10.1038/lsa.2016.96 (PMC6059946; doi:10.1038/lsa.2016.96)
Supplement: Supplementary Information [file lsa201696x1.docx]

Supplemental Information

**Controllable optical activity with non-chiral plasmonic metasurfaces**

Ping Yu^1^, Jianxiong Li^1^, Chengchun Tang^2^, Hua Cheng^1^, Zhaocheng Liu^1^, Zhancheng Li^1^, Zhe Liu^2^, Changzhi Gu^2^, Junjie Li^2,*^, Shuqi Chen^1,*^, and Jianguo Tian^1,*^

^1^The MOE Key Laboratory of Weak Light Nonlinear Photonics, School of Physics, Teda Applied Physics Institute, and the 2011 Project Collaborative Innovation Center for Biological Therapy, Nankai University, Tianjin 300071, China

^2^Beijing National Laboratory for Condensed Matter Physics, Institute of Physics, Chinese Academy of Sciences, P.O.Box 603, Beijing 100190, China

^*^Correspondence: SQ Chen, Email: [schen@nankai.edu.cn](mailto:schen@nankai.edu.cn); JG Tian, Email: [jjtian@nankai.edu.cn](mailto:jjtian@nankai.edu.cn); JJ Li, Email: jjli@iphy.ac.cn

**Supplementary Figures**


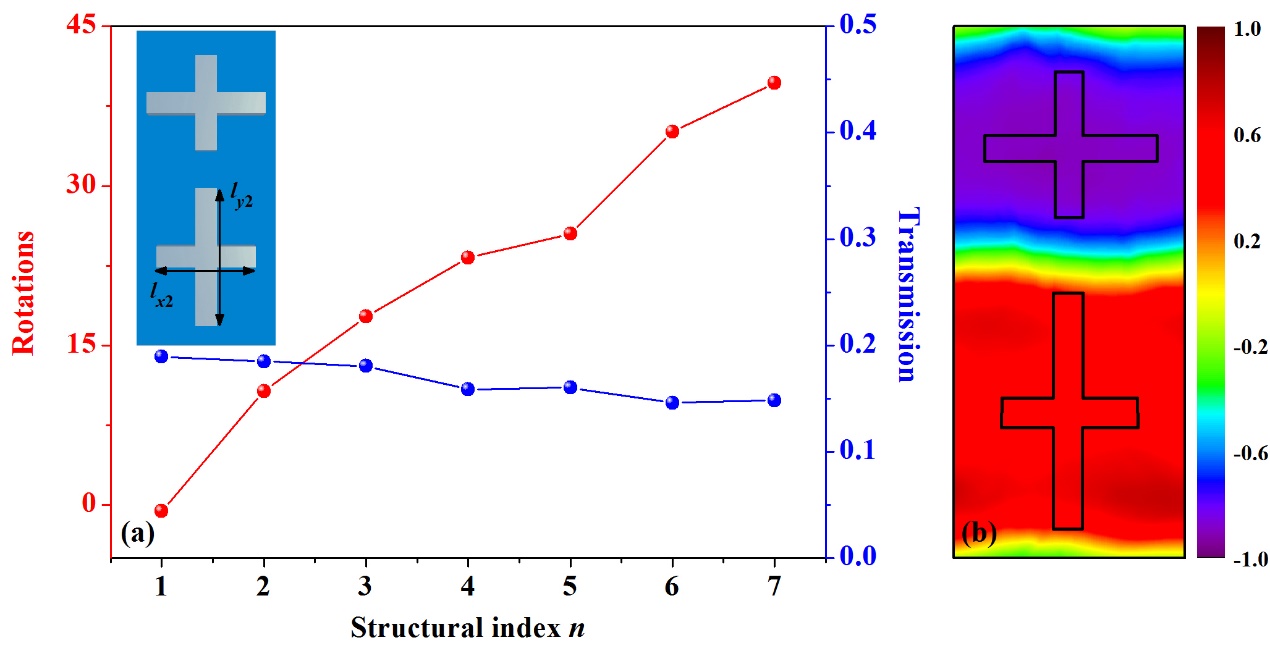


**Supplementary Figure S1.** **Non-chiral metasurface composed of cross-shaped nanoantennas** **(a)** Calculated optical rotations (red line) and amplitude transmissions (blue line) at a wavelength of 990 nm. Structural index *n* represents different parameters of (*l_x_*_2_, *l_y_*_2_), corresponding to (175, 280), (175, 260), (175, 265), (175, 255), (175, 245), (175, 235), and (175, 225) nm, for seven non-chiral cross-shaped nanoantennas. Insets show the unit cell of the non-chiral metasurface. **(b)** Distributions of **Stokes parameters *S*_3_ at 200 nm away from the exit surface of the** non-chiral **metasurface.**


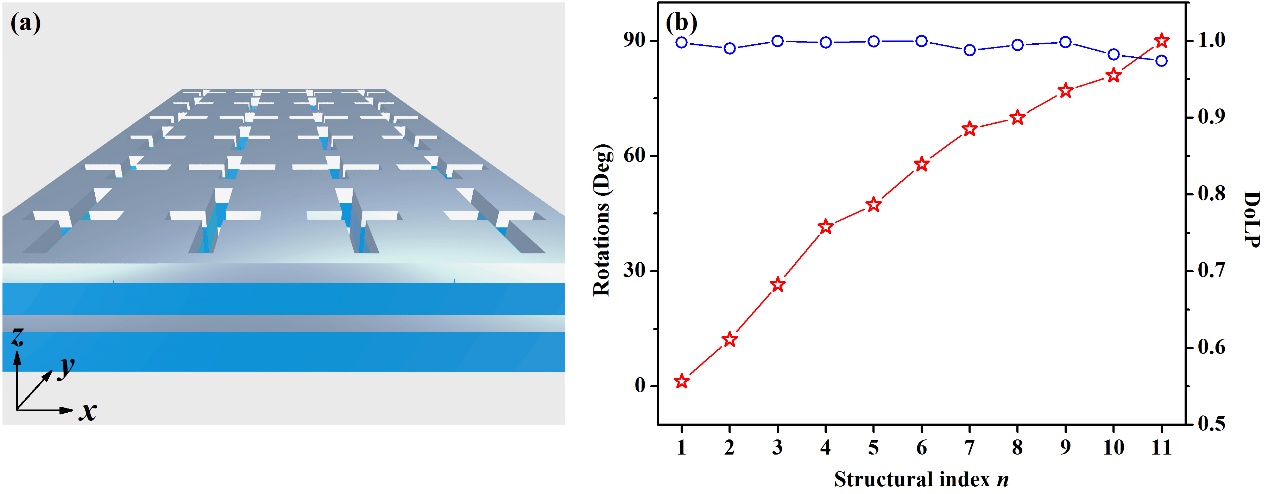


**Supplementary Figure S2.** **Large optical activity with dual-layer non-chiral metasurface.** **(a)** Schematic model of dual-layer non-chiral metasurface. **(b)** Calculated polarization optical rotations and degree of linear polarization (DoLP) of transmitted light at a wavelength of 1085 nm. Structural index *n* represents different parameters of (*l_x_*_2_, *l_y_*_2_), corresponding to (170, 330), (180, 340), (190, 350), (200, 370), (220, 370), (240, 330), (230, 310), (220, 300), (210, 290), (200, 280), and (190, 270) nm.


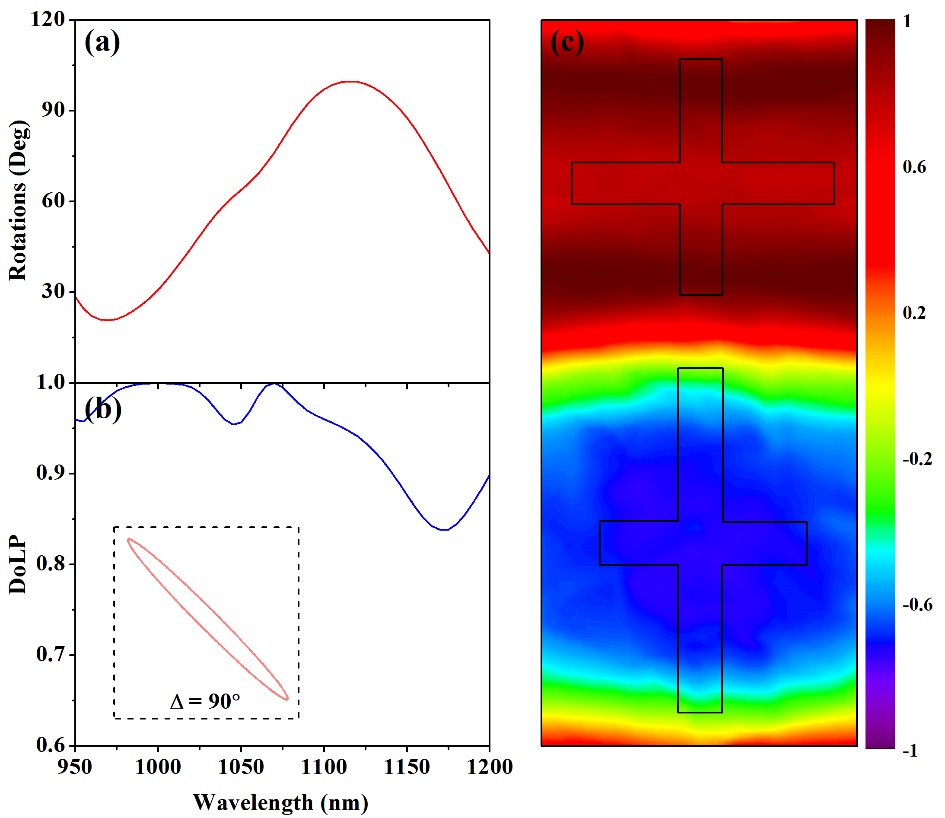


**Supplementary Figure S3. Characteristics of non-chiral dual-layer metasurface. (a)** Calculated optical rotations and **(b)** DoLP of the dual-layer metasurface that could realize 90° rotation. **(c)** The corresponding near field normalized Stokes parameters *S*_3_ at 220 nm away from the exit surface of the dual-layer metasurface.

**Supplementary Note 1: General applicability of the controllable optical activity** **with non-chiral metasurfaces.**

The proposed theory has extensive and general applicability, even to non-chiral nanoantennas. Here, we **consider the complementary geometry formed by** non-chiral **cross-shaped nanoantenna arrays. By applying Babinet’s principle to properly extend the plasmonic screens,^1^ we ensured that the non-chiral cross-shaped nanoantennas can also realize similar optical activity effects based on our proposed theory.** Figure S1a illustrates **the** rotations and corresponding transmissions at **the same** **wavelength** of 990 nm as that of the **non-chiral cross-shaped** nanoapertures. **The controllable optical activity is achieved by varying of the geometrical parameters *l_x_*_2_ and *l_y_*_2_, while the other geometrical parameters are the same as those of the cross-shaped nanoapertures. We also calculated the** **Stokes parameters *S*_3_ in the near field,** as shown in the Fig. S1b. **The distributions of the right circularly polarized (RCP) and left circularly polarized (LCP) lights are interchanged since the complementary nanostructures exhibit complementary field distributions.^2^ The proposed theory, therefore, can help to open the possibilities of realizing controllable optical activity by designing various kinds of non-chiral plasmonic metamaterials or metasurfaces.**

**Supplementary Note 2: Large optical activity by non-chiral dual-layer metasurfaces.**

**To further enhance the phase delay between the LCP and RCP lights, and to achieve a larger degree of optical activity, we adopted a** non-chiral **dual-layer plasmonic metasurface.** Figure S**2a shows the schematic model of the** non-chiral **dual-layer metasurface composed of two 50-nm-thick single-layer** cross-shaped nanoapertures**, which are separated by a 35-nm-thick SO_2_ layer.** The geometrical parameters of the cross-shaped nanoapertures in two layers are the same. Owing to the strong coupling between two layers, the non-chiral **dual-layer plasmonic metasurface exhibits more excellent ability to control the phase of transmitted light, leading to large optical activity of the incident** linearly polarized (LP) **light. The calculated rotation angle of the** LP **light is shown in** Figure **S2b. Results show that this angle can vary within a 90° range by varying the geometrical parameters** (*l_x_*_2_, *l_y_*_2_) **of the lower sub-units, while** fixing the geometrical parameters of the upper sub-units**. The corresponding** degree of linear polarization (DoLP) **shows that the transmitted light maintains nearly perfect linear polarization.**

Figures S3a and S3b illustrate the calculated spectra of the rotation and DoLP of the non-chiral dual-layer metasurface for 90° rotation. The effect of large optical activity can be observed at a wavelength of around 1085 nm. The transmitted phase difference between the LCP and RCP lights is modulated to 3*π*/2. The inset in Fig. S3b clearly shows the far-field polarization state of the transmitted light at 1085 nm. To confirm the proposed theory, the simulated normalized near-field distributions *S*_3_ are also given in Figure S3c. Owing to the existence of coupling between the two layers, the LP light will gradually be converted into LCP or RCP light after propagating a distance of more than 220 nm away from the exit surface. As with the case of non-chiral single-layer metasurface, the RCP and LCP lights are respectively obtained from the two sub-units, and these two rays effectively generate new LP light with 90° rotation at the far field. Thus, large optical activity can be further realized by designing the non-chiral dual-layer plasmonic metasurface.

**References**

1. Falcone F, Lopetegi T, Laso MAG, Baena JD, Bonache J *et al*. Babinet principle applied to the design of metasurfaces and metamaterials. *Phys Rev Lett* 2004; **93**: 197401.
2. Rockstuhl C, Zentgraf T, Meyrath TP, Giessen H, Lederer F. Resonances in complementary metamaterials and nanoapertures. *Opt Express* 2008; **16**: 2080.
